# Metabonomic Profiles Delineate the Effect of Traditional Chinese Medicine *Sini* Decoction on Myocardial Infarction in Rats

**DOI:** 10.1371/journal.pone.0034157

**Published:** 2012-04-06

**Authors:** Guangguo Tan, Wenting Liao, Xin Dong, Genjing Yang, Zhenyu Zhu, Wuhong Li, Yifeng Chai, Ziyang Lou

**Affiliations:** 1 School of Pharmacy, Second Military Medical University, Shanghai, China; 2 Shanghai Key Laboratory for Pharmaceutical Metabolite Research, Shanghai, China; Cardiovascular Research Institute Maastricht, Maastricht University, Netherlands

## Abstract

**Background:**

In spite of great advances in target-oriented Western medicine for treating myocardial infarction (MI), it is still a leading cause of death in a worldwide epidemic. In contrast to Western medicine, Traditional Chinese medicine (TCM) uses a holistic and synergistic approach to restore the balance of *Yin-Yang* of body energy so the body's normal function can be restored. *Sini* decoction (*SND*) is a well-known formula of TCM which has been used to treat MI for many years. However, its holistic activity evaluation and mechanistic understanding are still lacking due to its complex components.

**Methodology/Principal Findings:**

A urinary metabonomic method based on nuclear magnetic resonance and ultra high-performance liquid chromatography coupled to mass spectrometry was developed to characterize MI-related metabolic profiles and delineate the effect of *SND* on MI. With Elastic Net for classification and selection of biomarkers, nineteen potential biomarkers in rat urine were screened out, primarily related to myocardial energy metabolism, including the glycolysis, citrate cycle, amino acid metabolism, purine metabolism and pyrimidine metabolism. With the altered metabolism pathways as possible drug targets, we systematically analyze the therapeutic effect of *SND*, which demonstrated that *SND* administration could provide satisfactory effect on MI through partially regulating the perturbed myocardial energy metabolism.

**Conclusions/Significance:**

Our results showed that metabonomic approach offers a useful tool to identify MI-related biomarkers and provides a new methodological cue for systematically dissecting the underlying efficacies and mechanisms of TCM in treating MI.

## Introduction

Myocardial infarction (MI) has emerged as a major public health hazard. Evidence based medicine has resulted in the acceptance of nitrodilators, angiotensin converting enzyme inhibitors, angiotensin receptor blockers and anti-thrombotics as the standard treatment. In spite of great advances in drug treatment, it is still a leading cause of death in a worldwide epidemic [1], [2]. There is therefore an urgent need to discover new modalities of treatment for MI.

In contrast to target-oriented Western medicine, Traditional Chinese medicine (TCM) uses a holistic and synergistic approach to restore the balance of *Yin-Yang* of body energy so the body's normal function, or homeostasis, can be restored [3], [4]. *Sini* decoction (*SND*) is a representative TCM, which is officially recorded in Chinese pharmacopoeia 2010 edition and has been used to treat cardiovascular disease for many years [5], [6], [7]. It is composed of three medicinal herbs: *Acontium carmichaeli*, *Glycyrrhiza uralensis* and *Zingiber officinale*. Though many compounds have been isolated and identified from *SND* [8], evaluating the holistic efficacy and clarifying mechanism of the pharmacological action of *SND* remain a difficult task due to the mistiness of active compounds and the unknown synergistic actions of multiple components. Thus, new methods for activity evaluation and molecular target/pathway identification of such a multi-component medicine are sorely needed to advance the modernization of TCM.

Metabonomics is a top-down systems biology approach in which metabolic responses to biological interventions or environmental factors are analyzed and modeled [9], [10]. Metabonomics, monitoring entire pattern of low molecular weight compounds rather than focusing on individual metabolites, provides insights into the global metabolic status of entire organism, which is well coincident with the integrity and systemic feature of TCM [11], [12]. It has shown great promise to understanding disease mechanisms and identifying diagnostic biomarkers or drug targets [13], [14], [15], [16]. Analyzing the changes of metabolite profiles after treatment by TCM *in vitro* or *in vivo* may help dissect their underlying efficacies and mechanisms of action, and exploit new ideal drugs ultimately.

Nuclear magnetic resonance (NMR) spectroscopy and liquid chromatography-mass spectrometry (LC-MS) are the most frequently used analytical techniques in metabonomics [17], [18], [19]. Typically, either NMR or LC-MS is performed, but since these two techniques are complementary, the parallel use of these two techniques could achieve the most comprehensive screening of the entire metabolome. Wilson's group has illustrated that the two techniques applied in the same biofluid allowed different aspects of the metabolome to be investigated [20], [21], [22], [23].

In this work, ^1^H NMR and ultra high-performance liquid chromatography-mass spectrometry (UHPLC-MS) were used to generate metabolite profiles for the metabonomic analysis of urine collected from sham, MI model and *SND*-treated rats. One of the purposes is to characterize the metabolic changes-related to MI from NMR and UHPLC-MS analysis to increase the understanding of MI. The other purpose is to assess the therapeutic effects of *SND* to dissect the mechanisms of *SND*.

## Results

### Echocardiographic and histological assessment

**Figure 1:**
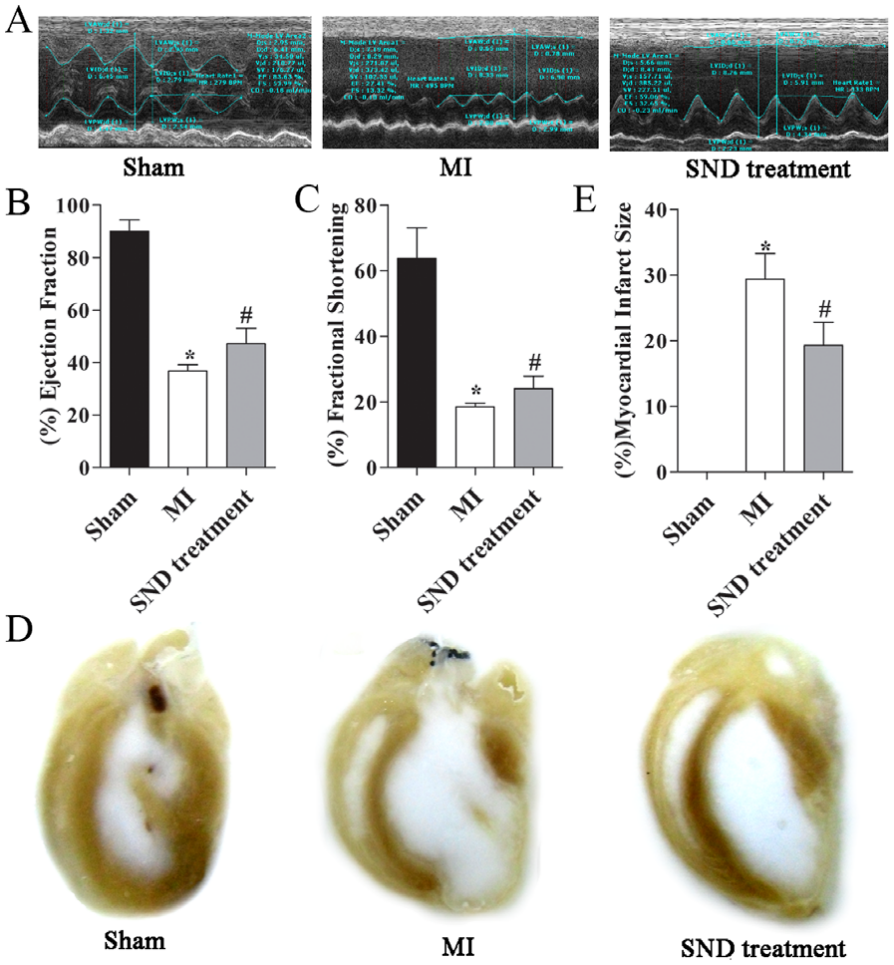
Echocardiographic and histological analysis. (A) M-mode echocardiography in rat models with sham operation, MI and *SND*-treated groups showing evidence of cardiac failure with chamber dilation in MI rat. *SND* prevented the development of chamber dilatation in MI rats. (B) Summary data for percentage of ejection fraction (EF) and (C) Summary data for percentage of fractional shortening (FS). Data shown are mean± S.D., n = 7, 6 and 8 for sham, MI, *SND*-treated groups, respectively. (D) Histologic sections of sham-operated and MI rat hearts, showing gross cardiac dilatation at the 21th day in the MI rats. Treatment of MI rats with *SND* prevented the development of cardiac remodeling. (E) Myocardial infarct size in sham, MI and *SND*-treated groups. No infarcted area appeared in sham group. Asterik (*) and pound key (#) represent statistically significant differences compared MI group to sham and *SND*-treated groups (*p*<0.05), respectively.

We assessed the systolic function in sham operated and MI rats compared to MI rats treated with *SND* using echocardiography. As shown in **Figure 1A** using two-dimensional and M-mode echocardiography, treatment with *SND* resulted in a significant improvement in left ventricle (LV) systolic function. Summary data for the ejection fraction and fractional shortening are shown in **Figure 1B** and **Figure 1C** as well as **Table 1** depicting a significant improvement in ejection fraction and fractional shortening in MI rats treated with *SND* at 21 days of follow up compared to MI alone.

**Table 1:** Summary of echocardiographic data.

Treatment	n	LVEDV(µL)	LVESV(µL)	LVIDd(mm)	LVIDs(mm)	EF(%)	FS(%)
Sham	7	170.94±35.26	17.91±8.85	5.87±0.55	2.16±0.66	90.02±4.24	63.78±9.23
MI alone	6	393.34±28.72	248.53±19.02	8.81±0.90	7.18±0.74	36.80±2.35*	18.52±1.11*
MI+SND	8	355.94±55.05	187.89±33.69	8.13±0.50	6.18±0.43	47.19±5.89#	23.99±3.92#

LVEDV, left ventricular end-diastolic volume; LVESV, left ventricular end-systolic volume; LVIDd,left ventricular internal diameter in diastole; LVIDs, left ventricular internal diameter in systole; EF, ejection fraction; FS, fractional shortening. Data are mean ± S.D.,

* *p*<0.05 comparing MI and sham animals,

# *p*<0.05 comparing *SND*-treatment and MI animals.

**Figure 1D** shows photomicrographs of examples of tissue sections from MI rats treated with *SND* for 21 days compared to MI alone or sham-operated hearts after 21 days of follow up. The MI rats showed evidence of an increase in chamber dilatation associated with MI at follow up. In contrast, treatment with *SND* prevented the development of cardiac dilatation post MI. The histopathological studies were also confirmed by myocardial infarct size evaluation. As shown in **Figure 1E**, compared to the MI group (29.4±3.91%), the infarct areas of the *SND*-treated group (19.3±3.50%) were significantly decreased (*p*<0.01). The above results indicated that the MI model was successfully established and the *SND* treatment had a therapeutic effect on MI.

### Urinary metabolite profiling in sham and MI rats by ^1^H NMR

**Figure 2:**
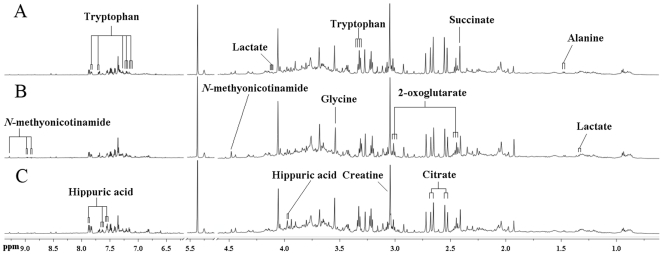
Representative 600 MHz ^1^H NMR spectra of urine from sham (A), MI (B) and *SND*-treated (C) groups.

**Figure 3:**
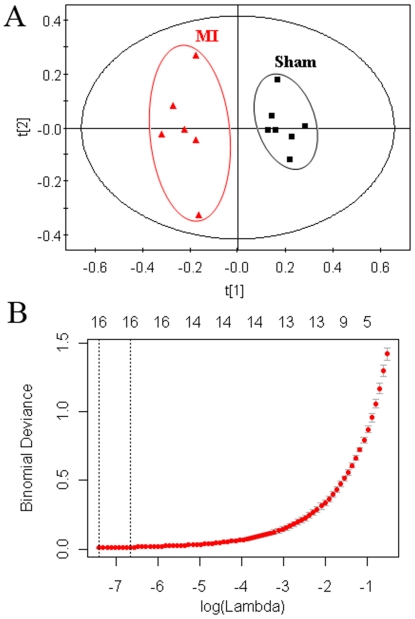
Multivariate statistical analysis of data from the ^1^H NMR analysis of urine from sham and MI groups. (A) PCA scores plot: (▪) sham group, (▴) MI group. (B)The relationship between lambda and deviance using Elastic Net approach (alpha = 0.8).

Typical ^1^H NMR spectra of urine samples collected on the 21th day from different groups are shown in **Figure 2**. Resonance assignment was carried out according to the literatures [24], [25] and an in-house database as well as the Metabonomics Toolbox (http://www.hmdb.ca). Due to the high information content and complexity of the spectra, multivariate data analysis was applied to reveal the metabolic changes-related MI. Initially, the unsupervised principal component analysis (PCA) was applied to explore correlations between sham and MI groups, and a tendency in the score plot to separate the two classes was detected (*R* ^2^ = 0.48) (**Figure 3A**). To further search features that can discriminate between groups, a unique challenge is posed because the number of variables is much bigger than the number of observations. The traditional partial least squares (PLS) method has extensively applied to selection of biomarkers in the similar metabonomic data [26]. Unfortunately, it cannot automatically retain good features. In this study, a recent developed approach, Elastic Net, was used for biomarker selection. The elastic net simultaneously does automatic variable selection and continuous shrinkage, and it can select groups of correlated variables [27]. It is like a stretchable fishing net that retains ‘all the big fish’. Thus, a variable list that can successfully discriminate the classes could be automatically obtained. **Figure 3B** shows the relationship between lambda and deviance using the Elastic Net approach for the 193 variables with 4-fold cross-validation. We can found that there was a small deviance for classification when we selected specific lambda value, an important parameter in Elastic Net, which can produce a classifier with 15 variables plus an intercept term. Thus, 15 variables were first selected as the candidates of potential biomarkers. Some of these segments were found to be from the same metabolites. After merging the variables from the identical metabolites, 10 metabolites were collected and considered as the potential biomarkers (**Table 2**).

**Table 2:** Identification of significantly differential metabolites in the rat urine by ^1^H NMR analysis.

Chemical shift (ppm)^a^	Metabolite	MI group^b^	SND group^c^	related pathway
1.34 (d), 4.10 (q)	Lactate	(*)↑	(*)↓	Glycolysis
1.50 (d)	L-alanine	(*)↑	(*)↓	Alanine and aspartate metabolism
2.42 (s)	Succinate	(*)↑	(*)↓	Citrate cycle
2.46 (t), 3.02 (t)	2-oxoglutarate	(*)↓	(*)↑	Citrate cycle
2.54 (d), 2.66 (d)	Citrate	(*)↓	(*)↑	Citrate cycle
3.06 (s)	Creatine	(*)↓	(*)↑	Arginine and proline metabolism
3.58 (s)	Glycine	(*)↑	(*)↓	Glycine, serine and threonine metabolism
7.18 (t), 7.34 (s)	L-tryptophan	(*)↓	(*)↑	Tryptophan metabolism
3.98 (d), 7.82(dd)	Hippuric acid	(*)↓	(*)↑	Phenylalanine metabolism
4.46 (s),9.30 (s)	*N*-methylnicotinamide	(*)↑	(*)↓	Tryptophan metabolism

a Letters in parentheses indicate the peak multiplicities: s, singlet; d, doublet; t, triplet; dd, doublet of doublets; q, quarte; ABX, two coupling.

b compared to sham group.

c compared to MI group. Arrow (↑) indicates relative increase in signal, Arrow (↓) indicates relative decrease in signal, Asterik (*) represents a statistically significant difference(*p*<0.05).

### Urinary metabolite profiling in sham and MI rats by UHPLC-MS

**Figure 4:**
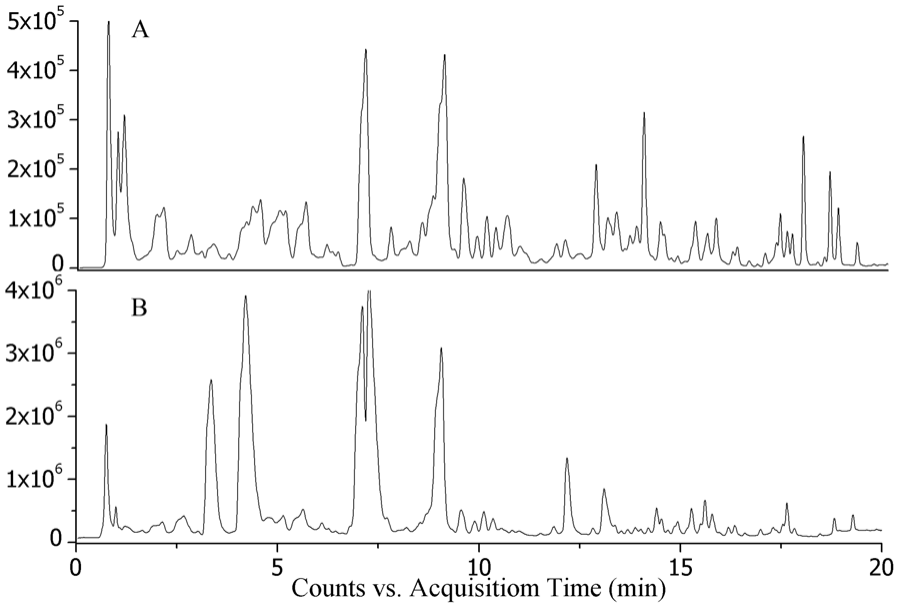
Typical urinary base peak intensity (BPI) chromatograms of sham rats in (A) ESI^+^ mode and (B) ESI^−^ mode.

**Figure 4** shows typical LC–MS total ion current (TIC) chromatograms of a urine sample in positive ionization mode (**Figure 4A**) and negative ionization mode (**Figure 4B**). To examine the stability and repeatability of the method, quality control (QC) samples were prepared by pooling the same volume of urine from all samples studied [28], [29]. System stability was evaluated by analysis of a QC sample six times at the beginning of the batch and then after every three samples. Six common ions in positive ion mode and in negative ion mode were selected for method validation, respectively. The result was 5.32%–10.37% for positive ion mode and 4.78%–9.58% for negative ion mode, which indicates that the method has good stability.

**Figure 5:**
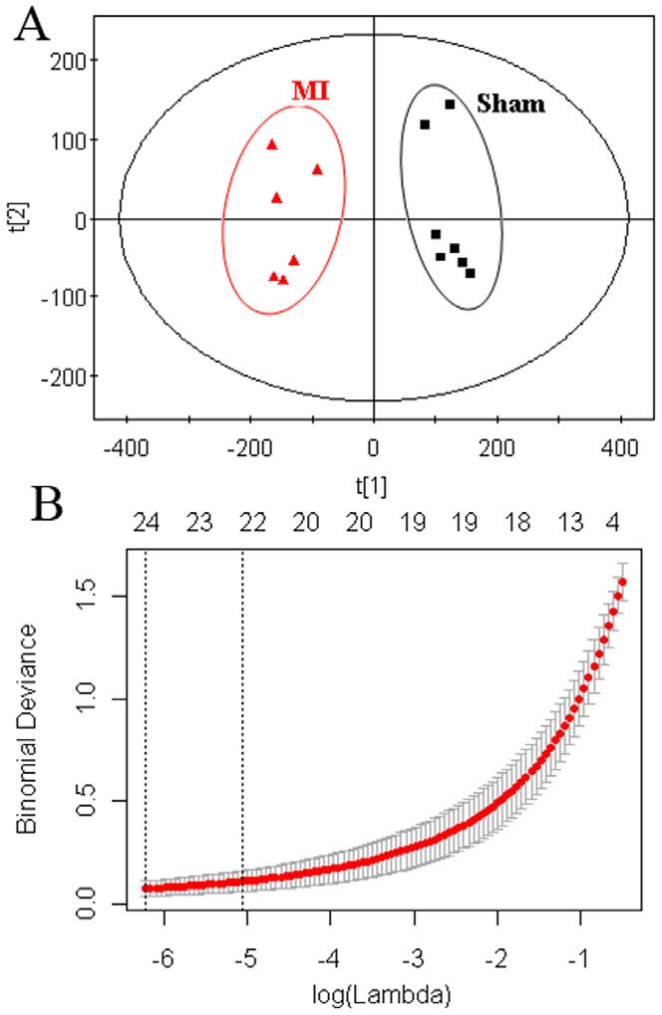
Multivariate statistical analysis of data from the ^1^H NMR analysis of urine from sham and MI groups. (A) PCA scores plot: (▪) sham group, (▴) MI group. (B)The relationship between lambda and deviance using Elastic Net approach (alpha = 0.8).

**Figure 6:**
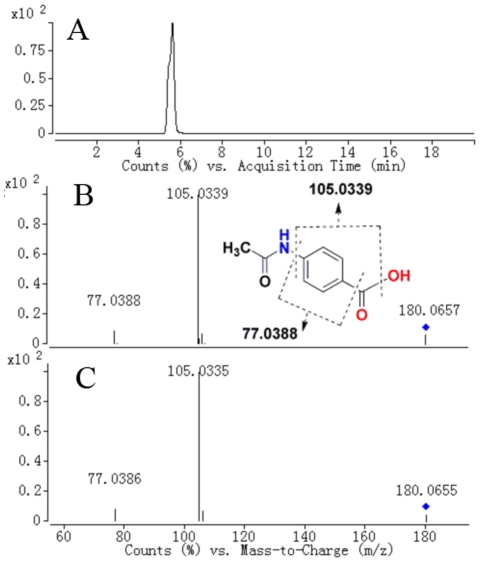
Identification of a selected marker (*m/z* 180). (A) Extracted ion chromatogram (EIC) of *m/z* 180; (B) MS/MS spectrum of the ion; (C) MS/MS spectrum of a commercial standard hippuric acid. The collision energy was 15 V.

The raw LC–MS data from metabolic profiling were pretreated following the procedure described in Section 2.8. Finally, 1345 ions, including ESI^+^ and ESI^−^ ions, were obtained. Similarly, PCA and Elastic Net were also applied to LC-MS dataset. In the PCA scores plot (**Figure 5A**), the MI group and sham group can also be well distinguished (*R* ^2^ = 0.57). **Figure 5B** shows the relationship between lambda and deviance using the Elastic Net approach for the 1345 variables with 4-fold cross-validation. It was found that there was a small deviance for classification when we selected specific lambda value, which can produce a classifier with 23 variables plus an intercept term. Thus, 23 variables were first selected as the candidates of potential biomarkers. With further checking the raw chromatograms, it was found that two sodium adduct ions of them were from the same metabolites. Finally, 21 metabolites were collected and considered as the potential biomarkers. Here, we take the ion at *m/z* 180 ([M+H]^+^)as an example to illustrate the identification process. First, the corresponding quasi-molecular ion peak was found according to the retention time in the extracted ion chromatogram (EIC) of *m/z* 180 (**Figure 6A**). The exact mass of the quasi-molecular ion was found as *m/z* 180.0658. Second, the element composition of the peak was calculated by Agilent MassHunter software. The calculated list provided 6 possible element compositions according to the exact mass and isotope pattern. Third, the elemental composition was compared to those registered in the freely accessible databases of HMDB (http://www.hmdb.ca), METLIN (http://metlin.scripps.edu) and KEGG (http://www.kegg.jp), and C_9_H_9_NO_3_ was found as the most likely compound. Fourth, a mass fragmentation experiment was conducted, in which two major fragment ions were found at *m/z* 105.033 and 77.038, which represent the fragments of [C_7_H_5_O]^+^ and [C_6_H_5_]^+^, respectively. The standard MS/MS spectrum of possible compounds was then matched. As a result, the biomarker was identified as hippuric acid and was finally confirmed by comparison with a standard compound (**Figure 6B** **and** **Figure 6C**). In this way, 13 metabolites (about 62% of the all) have been identified and listed in **Table 3**. However, the remaining biomarkers (data not shown) were unidentifiable due to insufficient intensity for MS/MS experiments or the restrictions of current metabolite databases.

**Table 3:** Identification of significantly differential metabolites in the rat urine by UHPLC-MS analysis.

No.	t_R_ (min)	Ion (*m/z*)	Metabolite	Ion mode	MI group^a^	SND group^b^	Related pathway
1	0.74	132.0769	Creatine^c^	ESI^+^	(*)↓	(*)↑	Arginine and proline metabolism
2	0.96	245.0771	Uridine^c^	ESI^+^	(*)↑	(*)↓	Pyrimidine metabolism
3	0.99	191.0195	Citrate^c^	ESI^−^	(*)↓	(*)↑	Citrate cycle
4	1.13	116.0710	L-proline^c^	ESI^+^	(*)↑	(#)↓	Arginine and proline metabolism
5	1.17	153.0656	4-PY or 2-PY^d^	ESI^+^	(*)↓	(#)↓	Nicotinate and nicotinamide metabolism
6	1.23	132.1018	L-isoleucine^c^	ESI^+^	(*)↓	(*)↑	Valine, leucine and isoleucine degradation
7	2.72	188.9865	Oxalosuccinate^c^	ESI^−^	(*)↓	(*)↑	Citrate cycle
8	3.83	205.0970	L-tryptophan^c^	ESI^+^	(*)↓	(*)↑	Tryptophan metabolism
9	5.59	180.0657	Hippuric acid^c^	ESI^+^	(*)↓	(*)↑	Phenylalanine metabolism
10	7.07	328.0420	Cyclic AMP^d^	ESI^−^	(*)↑	(*)↓	Purine metabolism
11	7.11	192.0666	Phenylacetylglycine^d^	ESI^−^	(*)↑	(*)↓	Phenylalanine metabolism
12	8.77	233.0920	N2-Succinyl-L-ornithine^d^	ESI^+^	(*)↓	(#)↑	Arginine and proline metabolism
13	9.04	283.0827	Xanthosine^c^	ESI^−^	(*)↓	(*)↑	Purine metabolism

a compared to control group.

b compared to MI group.

c Metabolites identified by comparing with database and authentic standard.

d Metabolites identified by comparing with literatures and database resources. Arrow (↑) indicates relative increase in signal. Arrow (↓) indicates relative decrease in signal. Asterik (*) represents a statistically significant difference (P<0.05), whereas pound key (#) represents no statistically significant difference. 4-PY: N-methyl-4-pyridone-3-carboxamide, 2-PY: N-methyl-2-pyridone-5-carboxamide.

### Effects of *SND* based on metabolite profiling

**Figure 7:**
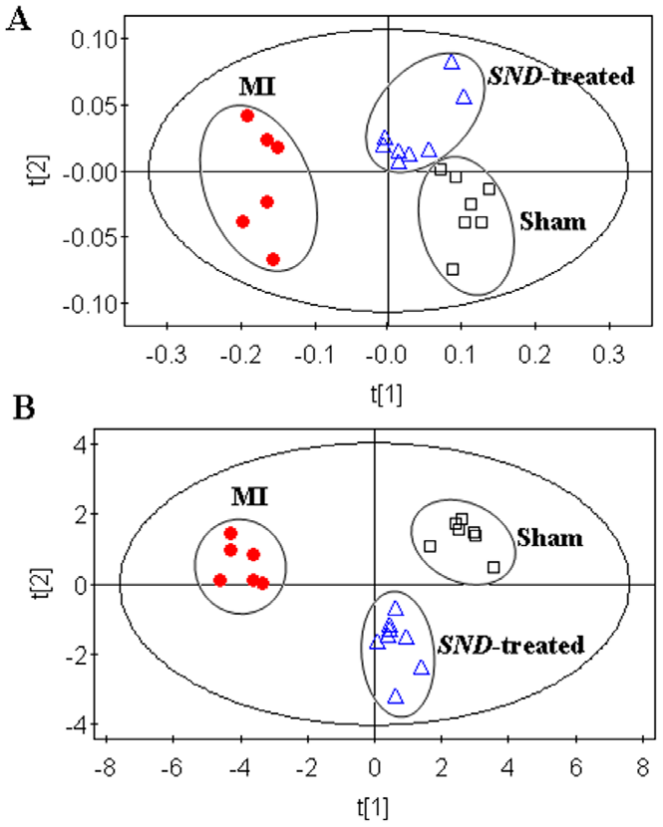
PCA scores plot derived from urine levels of ten metabolites using ^1^H NMR (A) and thirteen metabolites using UHPLC-MS (B) in sham group(▪), MI group(•) and *SND* treatment group (▴).

**Figure 8:**
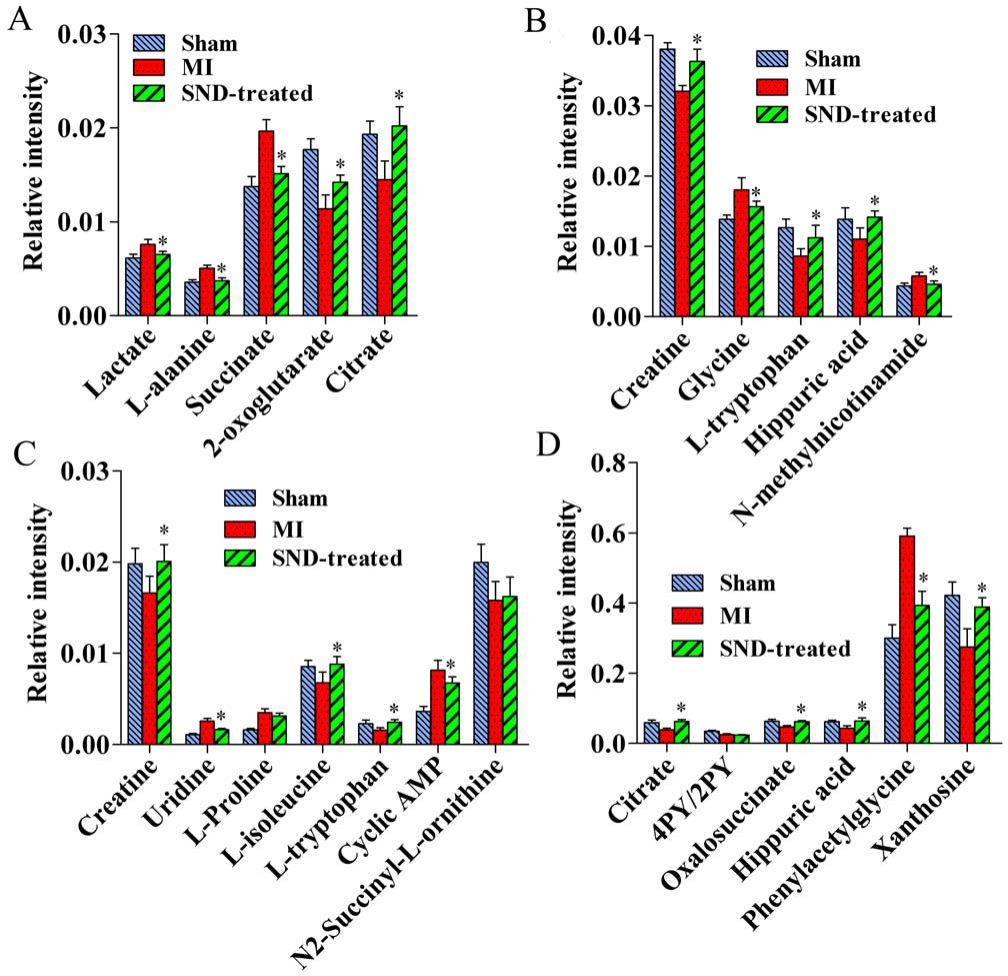
Bar plots show ^1^H NMR relative signal intensities for ten metabolites (A, B) and UHPLC-MS relative signal intensities for thirteen metabolites (C, D) in sham, MI and *SND*-treated groups. Data are expressed as mean ± S.D. Significant differences (*p*<0.05) exist between sham and MI for all the metabolites studied.*p<0.05 versus MI group.

Using the presented ^1^H NMR and UHPLC-MS method, the urinary metablic profiles of *SND*-treated group were obtained. The therapeutic effects of *SND* on MI have been shown in Section 3.1. As the 10 potential biomarkers from ^1^H NMR and 13 ones from UHPLC-MS have been found, it is reasonable to take them as the potential drug targets for further investigating the intervening mechanisms of *SND* to MI. Therefore, the levels of the 10 biomarkers from ^1^H NMR and the ones of 13 biomarkers from UHPLC-MS on the 21th day were introduced as variables to PCA, respectively, performed on sham, MI and *SND*-treated groups. The score plots of the first two principal components allowed visualization of the data and comparing of the three group samples. The *R^2^X* and *Q^2^* were 0.793 and 0.601 for ^1^H NMR and 0. 767 and 0.599 for UHPLC-MS, which indicated the classifications were well for PCA models with the variables detected by the respective techniques. As shown in the PCA scores map derived from urinary levels of ten metabolites using ^1^H NMR (**Figure 7A**), the *SND*-treated group is closer to the sham group. Similar result can be found from the PCA scores map derived from urinary levels of 13 metabolites using UHPLC-MS (**Figure 7B**). These results suggested that *SND* could reverse the pathological process of MI. To further evaluate the reversed condition of the potential biomarkers by administration with *SND*, student's t-test was performed by SPSS software. The critical *p*-value was set to 0.05 for significantly differential variables in this study. The relative peak areas of the 10 metabolites from ^1^H NMR and 13 metabolites from UHPLC-MS to their respective total integrated area of the spectra are shown in **Figure 8**. Compared to the MI group from ^1^H NMR dataset (**Figure 8A** **and** **Figure 8B**), ten metabolites including lactate, L-alanine, succinate, 2-oxoglutarate, citrate, creatine, glycine, L-tryptophan, hippuric acid, and *N*-methylnicotinamide were significantly reversed in *SND*-treated group. Similarly, from UHPLC-MS dataset (**Figure 8C** **and** **Figure 8D**), the ten of thirteen metabolites including creatine, uridine, citrate, L-isoleucine, oxalosuccinate, L-tryptophan, hippuric acid, cyclic AMP, phenylacetylglycine, and xanthosine were significantly reversed, and the other three metabolites were also reverse at different degrees, except for 4-PY (or 2-PY).

## Discussion

TCM has been used in China and other Asian countries for over 5,000 years for the prevention and treatment of a variety of diseases. A lot of clinical drugs for cardiovascular diseases are derived from natural products nowadays. Among which, different formulas of herbal medicines have been employed for treating MI based on TCM theory and empirical education. The TCM *SND* with long history of use has been proven to be effective in treating MI. Nevertheless, its holistic activity evaluation and mechanistic understanding are still lacking.

According to the chemical analysis of *SND* in our previous study [8], we found that the major components from *SND* are *aconitum* alkaloids, gingerols, flavonoids, and saponins. It was reported that *aconitum* alkaloids can positively influence heart related diseases when administered in optimum doses [30]. Gingerols have a direct positive inotropic effect and directly activates SR Ca^2+^-ATPase in mammalian myocardium [31], [32]. Several flavonoids and saponins were also reported to have cardiovascular activities [33]. For example, Isoliquiritigenin has a vasorelaxant effect [34] and glabridin can modulate vascular injury and atherogenesis [35]. Glycyrrhizin has an antiplatelet aggregation effect [36]. In addition, gingerols, flavonoids and saponins have antioxidant effects [33], [37]. Just as the multi-components hit multiple targets to exert an overall therapeutic effect, it is a great challenge to characterize the holistic efficacy and understand its action mechanisms completely.

**Figure 9:**
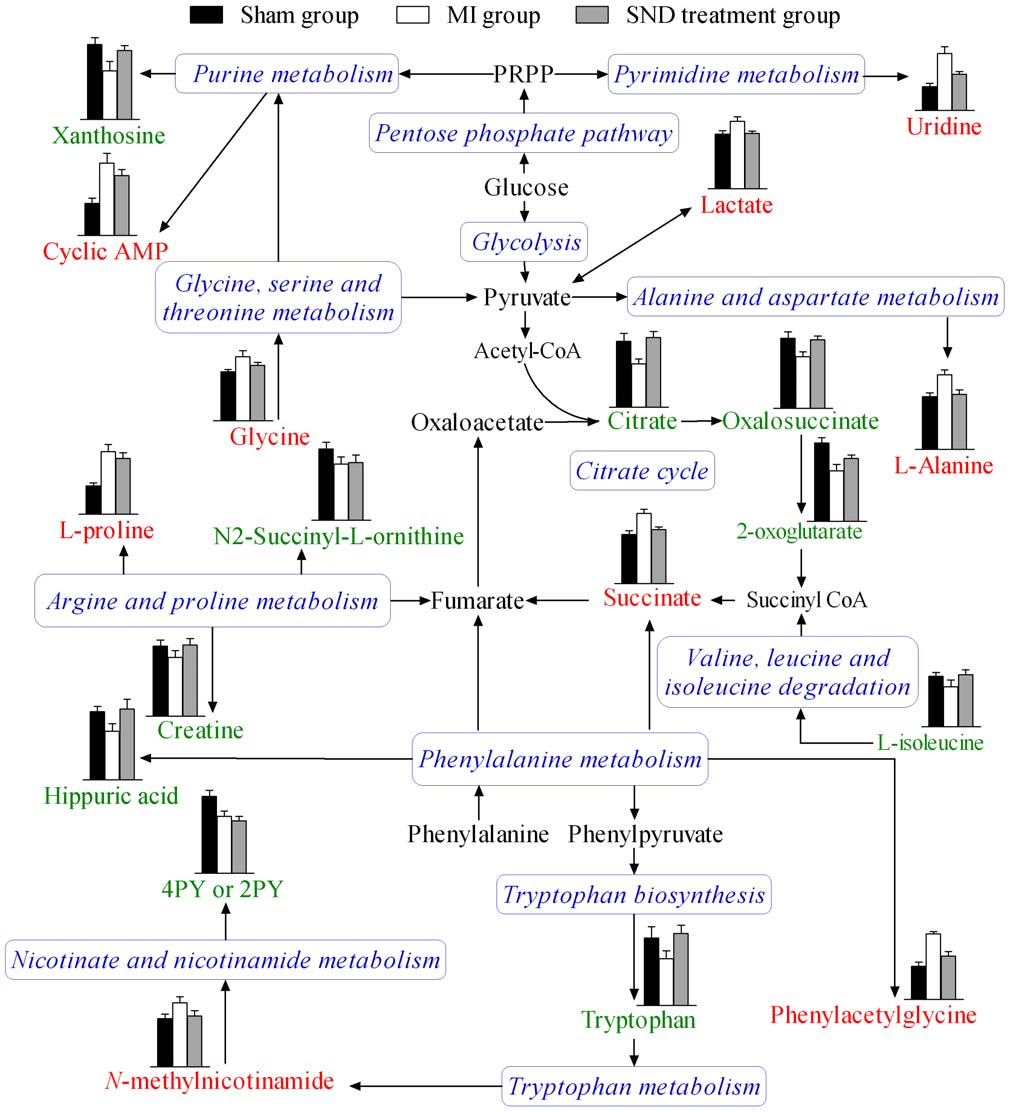
The network of the potential biomarkers changing for MI and *SND* modulation according to the KEGG PATHWAY database. Column value in histograms is expressed as mean ± S.D., in which the value of citrate, creatine, tryptophan and hippuric acid derived from UHPLC-MS and those of the other metabolites derived from respective analysis system (UHPLC-MS or ^1^H NMR). Metabolite names in red and green represent elevation and inhibition, respectively. Metabolite names in black mean they were not detected in our experiment. The blue italic words are pathway's names. PRPP, phosphoribosyl pyrophosphate.

In this study, both ^1^H NMR and UHPLC-MS were used to investigate the urinary metabolic profile associated with MI and then delineate the effect of TCM *SND* on MI. Although the changes in metabolite profiles observed by ^1^H NMR and UHPLC-MS with pattern recognition tools were similar, the markers which indicated the separations of MI group from sham group observed were different, apart from citrate, creatine, L-tryptophan and hippuric acid, which were detected using both techniques. It demonstrated that the potential biomarkers revealed by the two techniques were supplementary, which offered the potential to assess the perturbed metabolic pathways related to MI. Ultimately, nineteen potential biomarkers were identified and they distributed in 12 pathways by searching KEGG PATHWAY Database (http://www.genome.jp/kegg/). By relating the metabolic pathways, the metabolic network of the potential biomarkers changing for MI and *SND* modulation is constructed and shown in **Figure 9**.

Through the examination of **Figure 9**, we found that most of the potential biomarkers were involved in metabolic processes related to myocardial energy metabolism, including the glycolysis, citrate cycle, amino acid metabolism, purine metabolism and pyrimidine metabolism. Among which, the citrate cycle is central to energy metabolism in this network. Under the condition of MI, ischemia and oxygen deficiency resulted in reduced formation of adenosine triphosphate (ATP) via aerobic mechanisms and accelerated anaerobic ATP production by glycolysis [38], [39], [40]. Therefore, a build-up of lactate, the end product of anaerobic or nonoxidative glycolysis, was observed in MI group. It has also been used as a marker of ischemia in clinical practice and experimental studies [41], [42]. Meanwhile, the metabolite profiles showed the changes of four pivotal intermediates of citrate cycle, in which a decrease in citrate, oxalosuccinate, and 2-oxoglutarate and a build-up in succinate were observed in MI group. Cardiomyocyte levels of citrate cycle intermediates are tightly regulated to ensure adequate throughput of substrates derived from glycolysis and fatty acid oxidation [43]. The result indicated that the citrate cycle was inhibited, which is compatible with the results of previous studies [39]. The reason for its inhibition was related to the deficient oxygen and other substrate supply in mitochondrion.

A decrease in L-isoleucine in the MI group in comparison to the sham group was observed. L-Isoleucine is one of three branched chain amino acids (BCAAs, the others are leucine and valine). In myocardial ischemia, BCAAs derived from the mobilization of muscle protein may be an important alternative energy substrate for the heart [44]. It seems that the MI induced reduction of ATP production by inhibition of citrate cycle and fatty acid oxidation provoke the utilization of BCAA as an energy reservoir. The metabolite profiles also showed the changes of other α-amino acids metabolism, where a build-up in L-alanine and glycine as well as L-proline and a decrease in L-tryptophan were observed in MI group. They are important energy metabolism precursors and can be transformed into some biomolecules, such as pyruvate and fumarate, to enter into citrate cycle. One possible explanation was that ischemia and oxygen deficiency lead to their metabolic remodeling to meet energy requirement in myocardium [45]. In addition, *N*-methylnicotinamide, a metabolite of L-tryptophan detected in this study, was elevated in MI group, which may further predict the abnormality in the L-tryptophan metabolism in MI rats.

Uridine has been identified as a marker of myocardial viability after coronary occlusion and reperfusion [46], which was confirmed in our study. One possible explanation for enhanced uridine accumulation is a compensatory increase in RNA synthesis in the ischemic myocardium due to inefficient protein synthesis [46]. The UHPLC-MS spectra also showed a build-up of cyclic AMP and a decrease of xanthosine. The two metabolites are both involved in purine metabolism, which was an important process in myocardial ischemia injury [47], [48]. Among them, Cyclic AMP is a metabolite of ATP. The reason for its accumulation in urine of MI rat is probably related to the degradation of ATP. In contrast, due to the depletion of ATP, its biosynthesis from biomolecules, such as xanthosine, was compensatorily elevated in response to MI. This could explain the decreased level of xanthosine in MI group. Furthermore, a decrease in creatine in the MI group in comparison to the sham group was observed. It seems that the decreased level of creatine in serum and urine is a characteristic of MI [49]. In addition, the level of N2-succinyl-L-ornithine, hippuric acid, phenylacetylglycine and 4-PY (or 2-PY) was also changed in MI rat urine. However, it was perplexing corresponding to MI. A further study on the mechanisms of their changes in MI rats is being carried out in our laboratory.

It was reported that *SND* could up-regulate B-cell lymphoma 2 (Bcl-2) protein and inhibit activation of Caspase-3 in ischemia-reperfusion cardiomyocytes, resulting in promoting mitochondrial function and reducing apoptosis, which might be responsible for the intervening effect of *SND* on ischemia injury [7]. In this study, the down-regulation of lactate, succinate, and L-alanine and up-regulation of citrate, oxalosuccinate, 2-oxoglutarate, and L-tryptophan were observed in *SND*-treated group compared with MI group, which implied that *SND* might functionally intervene in glycolysis and citrate cycle as well as amino acids metabolism. In fact, *SND* administration permitted the mean levels of all potential biomarker to reverse at different degrees, except for 4-PY (or 2-PY). Combined with the echocardiographic assay, it suggested that *SND* has unique characteristics for the effect on MI. The potential biomarkers revealed metabolic pathways (glycolysis, citrate cycle, and amino acids metabolism) might be involved in the intervening mechanism of *SND*.

In summary, the myocardial ischemia and oxygen deficiency would lead to high rate of glycolysis, lactate accumulation, inhibition of citrate cycle and other disturbed metabolism. This hypothesis can be confirmed by combination of ^1^H NMR and UHPLC-MS with pattern recognition techniques in this study. With Elastic Net for classification and selection of biomarkers, nineteen metabolites primarily involved in glycolysis, citrate cycle, amino acid metabolism, purine metabolism and pyrimidine metabolism, were screened out and considered as potential biomarkers corresponding to MI. The ^1^H NMR and UHPLC–MS-based urinary metabonomic profiling has been successfully applied to evaluate the intervening effect of *SND*. Taking the potential biomarkers found in this study as possible drug targets, it revealed that *SND* could restore the unbalanced myocardial energy metabolism. However, due to the small number of rats in this study, our results serve to demonstrate the methodology rather than to provide definitive conclusions about the disease and drug. Application of these tracers for the detection of MI-induced disturbed metabolite status and assessment of the holistic efficacy of *SND* in other species including humans awaits further study. Our results show that combination of high resolution analytical tools (e.g., ^1^H Nuclear Magnetic Resonance Spectroscopy and Liquid Chromatography/Mass Spectrometry) with pattern recognition techniques provided a new methodological cue for dissecting the underlying efficacies and mechanisms of TCM.

## Materials and Methods

### Ethics Statement

All animal experiments were approved by the Administrative Committee of Experimental Animal Care and Use of Second Military Medical University (SMMU, Licence No. 2011023), and conformed to the National Institute of Health guidelines on the ethical use of animals.

### Reagents and materials

Formic acid of HPLC grade was purchased from Tedia (OH, USA). Acetonitrile of HPLC grade was purchased from Merck (Darmstadt, Germany). Distilled water was purified using a Milli-Q system (Millipore, Bedford, MA, USA). Deuterium oxide (D_2_O, 99.9%) was purchased from Minipul, Norell Inc. (Landisville, NJ, USA). Oxalosuccinate and xanthosine were purchased from Sigma-Aldrich (St Louis, MO, USA). The following compounds were obtained from Shanghai Jingchun Reagent Co.: creatine, uridine, citrate, L-isoleucine, L-tryptophan, hippuric acid, sodium dihydrogen phosphate dihydrate (NaH_2_PO_4_·2H_2_O) and disodium hydrogen phosphate dodecahydrate (Na_2_HPO_4_·12H_2_O).

*Acontium carmichaeli* (collection in Sichuan, China), *Glycyrrhiza uralensis* (collection in Xinjiang, China) and *Zingiber officinale* (collection in Guizhuo, China) were purchased from Shanghai Dekang Medicine Corp. (Shanghai, China) were authenticated by *Lianna Sun* (Department of Pharmacognosy, School of Pharmacy, Second Military Medical University, Shanghai, China).

### Preparation of *SND*

According to the original composition of *SND* recorded in *Chinese Pharmacopoeia* 2010 edition, *SND* was prepared using the following procedure. The crude drugs of *A. carmichaeli* 90 g, *Z. officinale* 60 g and *G. uralensis* 90 g were immersed in 2.4 liter water for 1 h and then decocted to boil for 2 h. The decoction was filtered through four layers of gauze. Next, the dregs were boiled once again for 1 h with 1.9 liters of water and the decoction was filtrated out with the above method. Afterward, the successive decoctions were merged and condensed under decompression. Finally, the extraction solution was made to a concentration of 1.0 g crude drugs/mL. According to our previous published paper [8], 53 components of *SND* were identified.

### Myocardial infarction mode and drug administration

All of the animal studies followed the relevant national legislation and local guidelines and were performed at the Centre of Laboratory Animals of the Second Military Medical University (Shanghai, China). Twenty-four male Sprague-Dawley rats (200±15 g) were purchased from the Slac Laboratory Animal Co., LTD (Shanghai, China) and housed in standard conditions. Myocardial infarction was produced by occlusion of the left anterior descending coronary artery, as described previously [50]. Anterior thoracotomy was performed under sterile conditions to open the pericardium. The heart was then rapidly exteriorized. The left anterior descending coronary artery was ligated approximately 2–3 mm distal from its origin with use of a 6-0 polypropylene suture. 21 animals survived throughout the experiment, including 14 MI rats and 7 sham rats (without ligation), while 3 animals died after surgery and were excluded. Eight of 14 MI rats received *SND* by oral gavage at dose of 10 g/kg body weight (equal to 10 mL/kg body weight) once daily between 8:00 and 10:00 a.m. for the following 21 days. The sham (n = 7) and MI (n = 6) rats received the same volume of water vehicle. The physiological examinations were performed 21 days after heart surgery based on two points. On one hand, the functional deterioration after left coronary artery ligation is generally maximal after 3 weeks [51]. On the other hand, TCM to exert remarkable effects needs more time than western medicine based on TCM theory. The administration procedure in this study is in accordance with clinical use.

### Sample collection and preparation

Compared to serum metabonomic study, the urine metabonomic study is non-invasion. Therefore, we focus on MI-related urinary metabolites in this study and samples of 24-h urine were collected on the 21th day from sham, MI and *SND*-treaded groups. Sodium azide was added to the collection vessels as an antibacterial agent. The fresh urine samples were immediately centrifuged at 14,000×g for 10 min at 4°C, to remove particle contaminants, and the supernatants were stored at −80°C until NMR and UHPLC -MS analysis.

For ^1^H NMR analysis, an aliquot of 400 µL of urine sample was mixed with 200 µL phosphate buffer (0.2 M Na_2_HPO_4_ and 0.2 M NaH_2_PO_4_; pH 7.4), 50 µL TSP (3-trimethylsilyl-propionic acid; 1 mM final concentration; internal standard) and 50 µL deuterium oxide. The mixture was left to stand for 10 min at room temperature and then centrifuged at 14,000×g for 15 min at 4°C in order to remove any precipitates. The supernatant of 600 µL was transferred into 5-mm o.d. NMR tube.

For UHPLC-MS analysis, 400 µL of methanol was added to 100 µL aliquots of urine. The mixture was vortex-mixed vigorously for 30 s and subsequently centrifuged at 14,000×g for 15 min at 4°C. The supernatant was transferred to autosampler vial kept and an aliquot of 4 µL was injected for UHPLC-MS analysis.

### Echocardiography and histology

Echocardiography was performed with Visual Sonics Vevo 770 machine equipped with 23 (or 30) MHz transducers on one day post-sampling to assess systolic function. Animals were lightly sedated with 100 mg/kg ketamine (Anhui Wanhe Pharmaceuticals, China). Left ventricular end-systolic volume (LVESV), left ventricular end-diastolic volume (LVEDV), left ventricular internal diameter in diastole (LVIDd), and left ventricular internal diameter in systole (LVIDs) were measured at the level of the papillary muscles on the short-axis view using 2-dimensional guided M-mode imaging at 3 cardiac cycles. The left ventricular (LV) ejection fraction (EF) and fractional shortening (FS) were taken as measures of LV systolic function [52]. Left ventricular EF and FS were calculated using EF% = ((LVEDV−LVESV)/LVEDV)×100 and FS (%) = (LVIDd−LVIDs )/LVIDd×100, respectively.

Hearts were excised, and we randomly split the samples of each group in half, and use one half of the sample to roughly estimate chamber dilatation and the other half of the sample to determine myocardial infarct size. The hearts for estimating chamber dilatation were retrogradely perfused with phosphate-buffered solution to wash out blood and fixed in 4% paraformaldehyde. Hearts were then embedded in tissue OCT-freeze medium and the long-axis section was taken perpendicular to the horizontal axis. In order to determine myocardial infarct size, duplicate 1-mm mid-LV sections of the other frozen hearts were cut and incubated with 1% triphenyltetrazolium chloride for 20 min at 37°C. The infarct size was determined by planimetry of the infarct zone and expressed as a percentage of the total LV area using HPIAS-1000 color pathology picture analysis system.

### ^1^H NMR analysis

^1^H NMR analysis was made on a Bruker AVANCE II 600 spectrometer, operating at 600.13 MHz 1H frequency (Bruker Spectrospin AG, SWISS). The NMR spectra of the urine samples were acquired using a solvent pre-saturation pulse sequence to suppress the residual water resonance. Free induction decays (FIDs) were collected at 64 k data points, at 300 K, with a spectral width of 7200 Hz and an acquisition time of 2.04 s, giving a total pulse recycle delay of 3.04 s. The data were zero filled by a factor of 2 and the FIDs were multiplied by an exponential weighting function equivalent to a line broadening of 0.3 Hz prior to Fourier transformation.

### UHPLC-MS analysis

UHPLC-MS analysis was performed on Agilent 1290 Infinity LC system coupled to Agilent 6530 Accurate-Mass Quadrupole Time-of-Flight (Q-TOF) mass spectrometer (Agilent, USA). Chromatographic separations were performed on an ACQUITY UPLC™ BEH C_18_ column (2.1 mm×100 mm, 1.7 µm, Waters, Milford, MA) maintained at 50°C. The mobile phase consisted of 0.1% formic acid (A) and ACN modified with 0.1% formic acid (B). The following gradient program was used: 3%B at 0–2 min, 3%–15% B at 2–11 min, 15%–30% B at 11–16 min, 30%–95% B at 16–18 min, 95% B at 18–19.5 min, 95%-3%B at 19.5–20 min and followed by re-equilibrated step of 4 min. The flow rate was 400 µL/min and the injection volume was 4 µL.

An electrospray ionization source (ESI) interface was used, and was set in both positive and negative modes so as to monitor as many ions as possible. The following parameters were employed: capillary voltage, 3.5 kV; drying gas flow, 11 L/min; gas temperature: 350°C; nebulizer pressure, 45 psig. fragmentor voltage, 120 V; skimmer voltage, 60 V. Data were collected in centroid mode and the mass range was set at *m/z* 50–1000 using extended dynamic range. Potential biomarkers were analyzed by MS/MS. The collision energy was 15 V.

### Data processing

The acquired ^1^H NMR spectra were manually corrected for phase and baseline distortions within TOPSPIN (version 3.0, Bruker Biospin, Germany), and the chemical shifts were referenced to TSP at 0.0 ppm. Each ^1^H NMR spectrum was reduced into 220 integrated segments of equal width (0.04 ppm) corresponding to the region between 0.60 and 9.40 ppm using AMIX software (version 3.9.5, Bruker Spectrospin Ltd.). The spectral regions containing residual water (4.64–5.08 ppm) and urea resonances (5.56–6.20 ppm) was excluded from the analysis. All remaining segments (193 segments) of the spectra were then scaled to the total integrated area of the spectra in order to reduce any significant concentration differences. The resulting three-dimensional matrix, including spectral regions (variable indices), sample names (observations), and normalized integral intensities (variables), was exported to multivariate data analysis.

The acquired UHPLC-MS raw data in instrument specific format (.d) were firstly converted to common data format (.mzData) files using a conversion software program (file converter program available in Agilent MassHunter Qualitative software), in which the isotope interferences were eliminated. The program XCMS (http://metlin.scripps.edu/download/) was then used for nonlinear alignment of the data in the time domain and automatic integration and extraction of the peak intensities [53]. XCMS parameters were default settings except for the following: full width at half maximum (FWHM) = 10, bandwidth (bw) = 10 and snthresh = 6, due to narrower peaks obtained by the use of the column packed with 1.7 µm particles. The variables presenting in at least 80% of either group were extracted [54]. Variables with less than 30% relative standard deviation (RSD) in QC samples [28], [29] were then retained for further multivariate data analysis because they were considered stable enough for prolonged LC–MS analysis. For each chromatogram, the intensity of each ion was normalized to the total ion intensity, in order to partially compensate for the concentration bias of metabolites between samples and to obtain the relative intensity of metabolites. The resulting three-dimensional matrix, including retention time and m/z pairs (variable indices), sample names (observations), and normalized ion intensities (variables), was exported to multivariate data analysis.

### Statistical analysis

Statistically significant differences in mean values were tested by using 2-tailed, 2-sample Student's t-test, and *p*<0.05 was considered statistically significant.

Prior to multivariate analysis, the resultant data matrices from two analytical techniques were mean-centered and paretoscaled. The PCA using SIMCA-P (version 11, Umetrics) was used to uncover unknown trends in the sham, MI, and *SND*-treated groups. The Elastic Net using the library “glmnet” in the R environment (http://www.r-project.org) was used for classification and selection of significant ion peaks between sham and MI groups.
